# Supplementary material for: Pervasive distribution of polyester fibres in the Arctic Ocean is driven by Atlantic inputs
Source: Nat Commun. 2021 Jan 12;12:106. doi: 10.1038/s41467-020-20347-1 (PMC7804434; doi:10.1038/s41467-020-20347-1)
Supplement: Supplementary file 1 — Supplementary Information [file 41467_2020_20347_MOESM1_ESM.pdf]

Supplementary Information for "Pervasive distribution of polyester fibres in the Arctic Ocean driven by Atlantic inputs" by Peter S. Ross *et al.*

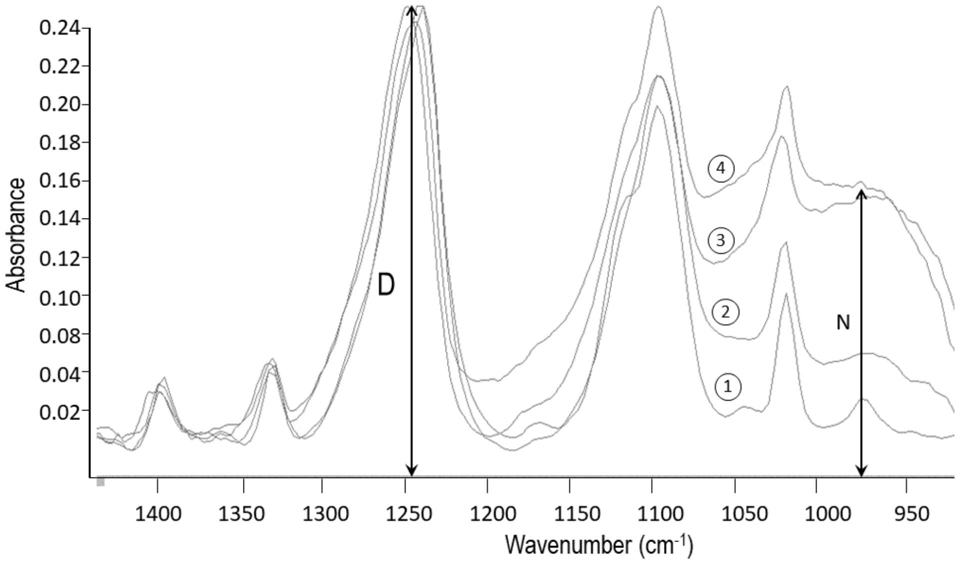

Supplementary Information Figure 1| Infra-red signatures appear to reflect environmental weathering. Differences in Infra-red signatures for polyester fibres were evident in our controlled pilot study of a new unweathered polyester textile sample (profile 1) compared to the same material incubated in the ocean over a period of one year (profile 2), and to two unknown fibres retrieved from seawater samples in the Arctic Ocean (profiles 3 and 4). The most prominent alteration was observed at 970 cm^{-1} (N), while peak 1241 cm^{-1} (D) was relatively consistent. The N:D ratio increased with the duration of controlled weathering and was used as a quantitative proxy for weathering of polyester fibres in the environment.

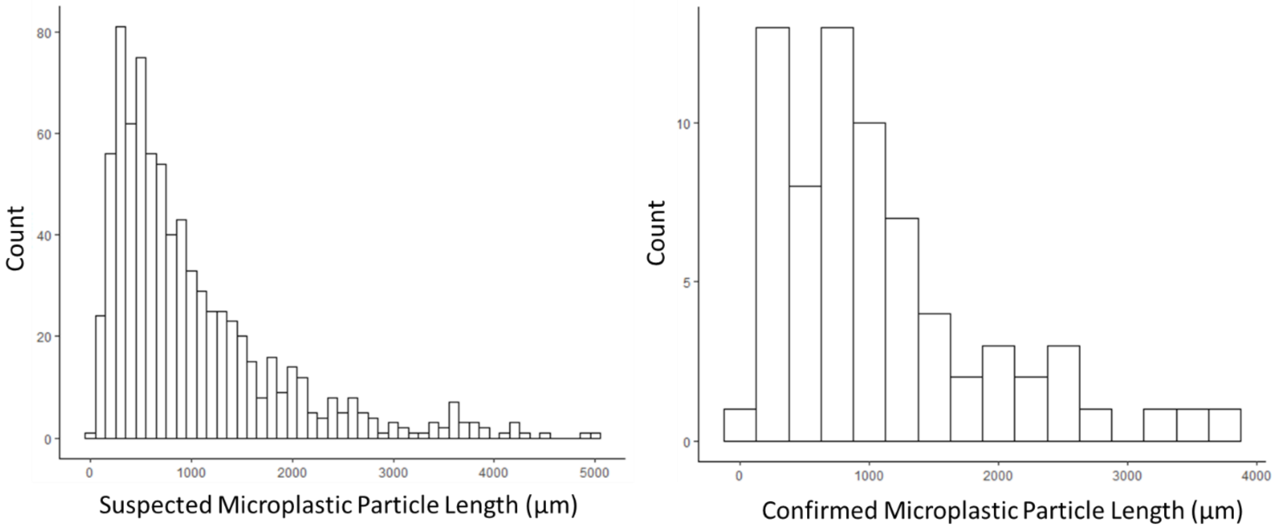

Supplementary Information Figure 2| Size frequency distributions for Suspected Microplastics (SMPs; left) and Microplastics (MPs) confirmed using Fourier Transform Infra Red Spectrometry (FTIR; right). Our use of a 63µ mesh size at sampling may have increased retention of MPs slightly relative to other studies employing a 330µ mesh size in the field, since 23% of SMPs and 19% of MPs fell below the 330µ micron mesh.

Supplementary Table 1 | Polyester fibres dominate microplastic profiles in Arctic seawater. Polyester (polyethylene terephthalate) fibres dominated confirmed MP particles in all Arctic samples (near surface and at depth), underscoring the extent to which this category of MPs has been dispersed. Data is based on the FTIR analysis of 422 of 1570 of suspect microplastics from all Arctic samples (near surface and depth).

Polymer	All MP particles	All MP fibres
Polyester	72.3%	73.3%
Acrylic	9.2%	8.3%
Nylon	7.7%	8.3%
Unknown plastics	6.1%	6.7%
Polypropylene	3.0%	3.3%
Polyvinyl chloride	1.5%	0%
Total	100%	100%

Supplementary Table 2 | Microplastic counts (a), lengths (b) and infra-red Peak Ratio Indices (c) for Figure 2.

2a) Counts

cruise	Station	Sample type	depth	volume	latitude	Longitude	region	Per m3
C3O	loop70	loopsieved	0	63.8	64.14	-171.725	chukchi	6.202559098
C3O	loop74	loopsieved	0	59.9	65.006	-168.472	chukchi	20.60862276
C3O	loop77	loopsieved	0	56.4	67.0505	-168.7232	chukchi	5.673758865
C3O	loop79	loopsieved	0	56.2	68.255	-167.121	chukchi	1.897983393
C3O	loop83	loopsieved	0	57.8	71.08	-162.22	chukchi	18.73719659
C3O	loop88	loopsieved	0	56.6	71.15	-163.41	chukchi	1.884570082
C3O	loop89	loopsieved	NA	NA	71.5315	-163.8542	chukchi	NA
C3O	loop90blank	loopsievedblank	0	66.1	71.541	-163.856	chukchi	14.26464571
JOIS16	1047-1049	Rosette	42.428	30.54	71.3685	-152.0653	chukchi	121.6665539
JOIS16	1055-1057	Rosette	5.443	30.54	71.3685	-152.0653	chukchi	84.65618532
JOIS16	1064-1066	Rosette	1014.92	30.54	72.5975	-144.7043	beaufort	19.94291433
JOIS16	1068-1070	Rosette	485.673	30.54	72.5975	-144.7043	beaufort	28.47664143
JOIS16	1072-1074	Rosette	265.07	30.54	72.5975	-144.7043	beaufort	39.72931674
JOIS16	1076-1078	Rosette	206.111	30.54	72.5975	-144.7043	beaufort	52.76893981
JOIS16	1080-1082	Rosette	60.157	30.54	72.5975	-144.7043	beaufort	10.47806156
JOIS16	1084-1086	Rosette	4.896	30	72.5975	-144.7043	beaufort	99.53789355
JOIS16	1136-1139	Rosette	1012.513	38.52	74.0023	-139.9428	beaufort	25.96053998
JOIS16	1140-1143	Rosette	496.567	38.52	74.0023	-139.9428	beaufort	15.07320939
JOIS16	1144-1147	Rosette	273.513	38.52	74.0023	-139.9428	beaufort	30.69548123
JOIS16	1148-1151	Rosette	217.656	38.52	74.0023	-139.9428	beaufort	7.504065519
JOIS16	1152-1155	Rosette	63.718	38.52	74.0023	-139.9428	beaufort	0
JOIS16	1156-1158	Rosette	5.04	28.64	74.0023	-139.9428	beaufort	260.5655371
JOIS16	548-550	Rosette	1003	30.54	78.0092	-149.8928	beaufort	34.44880702
JOIS16	553-555	Rosette	448.185	29.54	78.0092	-149.8928	beaufort	70.59975561
JOIS16	557-559	Rosette	255.444	30.54	78.0092	-149.8928	beaufort	44.68827355
JOIS16	560-562	Rosette	201.745	30.54	78.0092	-149.8928	beaufort	6.985374372
JOIS16	564-566	Rosette	44.366	30.54	78.0092	-149.8928	beaufort	66.0974575
JOIS16	568-570	Rosette	5.107	30.54	78.0092	-149.8928	beaufort	60.12529192
JOIS16	699-701	Rosette	1013.663	30.54	75.0027	-149.9902	beaufort	5.97216559
JOIS16	703-705	Rosette	515.708	30.54	75.0027	-149.9902	beaufort	111.8810229
JOIS16	707-709	Rosette	278.699	30.54	75.0027	-149.9902	beaufort	117.646377
JOIS16	711-713	Rosette	221.188	30.54	75.0027	-149.9902	beaufort	16.45022715
JOIS16	715-717	Rosette	61.09	29.34	75.0027	-149.9902	beaufort	13.4875007
JOIS16	719-721	rosette	5.451	29.34	75.0027	-149.9902	beaufort	43.93512624
JOIS16	764-766	rosette	20.755	30.44	74.9893	-150.023	beaufort	95.44677726
JOIS16	768-770	rosette	4.749	30.54	74.9893	-150.023	beaufort	119.5086284
JOIS16	A133	loopsieved	0	187	71.54048	-151.46474	chukchi	0.975347257
JOIS16	loop1	loopsieved	0	166.7	70.5643	-124.98682	chukchi	6.638672266
JOIS16	loop107	loopsieved	0	297.5	75.64318	-153.6673	beaufort	25.21802764
JOIS16	loop107blank	loopsievedblank	0	297.5	75.64318	-153.6673	beaufort	0.971618836
JOIS16	loop11	loopsieved	0	177.8	72.99048	-136.32976	beaufort	2.651555348
JOIS16	loop125	loopsieved	0	187	71.82197	-150.7859	chukchi	19.90900132
JOIS16	loop156	loopsieved	0	178.5	72.6174	-144.70258	beaufort	7.923471275
JOIS16	loop176	loopsieved	0	207.5	73.5898	-141.71382	beaufort	0.514056225
JOIS16	loop195	loopsieved	0	187	70.60314	-139.99664	chukchi	7.364065255
JOIS16	loop206	loopsieved	0	460.3	70.0691	-139.69424	chukchi	6.697827423
JOIS16	loop40	loopsieved	0	295.7	78.52782	-130.9431	beaufort	8.357952455
JOIS16	loop87	loopsieved	0	203.3	77.7518	-150.0257	beaufort	4.814806267
ONEOCEAN16	E01	loopsieved	0	30	69.2	-53.79	baffin	181.6765552
ONEOCEAN16	E02	loopsieved	0	30	70.61	-60.79	baffin	103.0934491
ONEOCEAN16	E03	loopsieved	0	30	71.55	-65.805	baffin	56.56934009
ONEOCEAN16	E04	loopsieved	0	30	72.55	-74.16	baffin	66.28812081
ONEOCEAN16	E05	loopsieved	0	30	72.698	-78.197	baffin	87.05211406
ONEOCEAN16	E06	loopsieved	0	30	73.44	-81	baffin	116.1248956

ONEOCEAN16	E07	loopsieved	0	30	74.488	-83.03	archipelago	84.52711452
ONEOCEAN16	E08	loopsieved	0	30	74.528	-85.447	archipelago	64.14166713
ONEOCEAN16	E09	loopsieved	0	30	74.531	-89.0363	archipelago	78.03743901
ONEOCEAN16	E10	loopsieved	0	30	74.703	-91.971	archipelago	62.32255431
ONEOCEAN16	E11	loopsieved	0	30	74.734	-92.039	archipelago	67.69722269
ONEOCEAN16	E12	loopsieved	0	30	74.017	-89.868	archipelago	15.7148847
ONEOCEAN16	E13	loopsieved	0	30	73.504	-90.569	archipelago	15.7148847
ONEOCEAN16	E14	loopsieved	0	30	72.006	-94.216	archipelago	204.5861116
ONEOCEAN16	E15	loopsieved	0	30	71.926	-95.626	archipelago	28.98922098
ONEOCEAN16	E16	loopsieved	0	30	71.789	-96.82	archipelago	22.90955641
ONEOCEAN16	E17	loopsieved	0	30	71.456	-97.031	archipelago	149.9116528
ONEOCEAN16	E18	loopsieved	0	30	69.665	-98.405	archipelago	55.53789355
UNCLOS	loop128	loopsieved	0	30	87.979	-90.57166667	pole	9.635220126
UNCLOS	loop135	loopsieved	0	30	88.538	-128.2518333	pole	24.31865828
UNCLOS	loop145	loopsieved	0	30	89.987	48.47566667	pole	77.95387841
UNCLOS	loop165	loopsieved	0	30	87.438	-134.7785	pole	33.33333333
UNCLOS	loop165blank	loopsievedblank	0	30	87.438	-134.7785	pole	32.54477653
UNCLOS	loop17	loopsieved	0	30	77.27667	8.8015	fram	80.88799837
UNCLOS	loop175	loopsieved	0	30	86.88	-142.5455	pole	55.37077234
UNCLOS	loop17blank	loopsievedblank	0	30	77.27667	8.8015	fram	143.2941133
UNCLOS	loop180	loopsieved	0	30	86.943	-138.2758333	pole	21.79454927
UNCLOS	loop190	loopsieved	0	30	86.371	-134.672	pole	44.70410568
UNCLOS	loop203	loopsieved	0	30	85.944	-147.8465	pole	107.3540009
UNCLOS	loop219	loopsieved	0	30	83.152	-141.9505	beaufort	26.46511196
UNCLOS	loop237	loopsieved	0	30	81.781	-140.5691667	beaufort	153.4237955
UNCLOS	loop237blank	loopsievedblank	0	30	81.781	-140.5691667	beaufort	9.635220126
UNCLOS	loop255	loopsieved	0	30	81.64	-146.1848333	beaufort	18.23899371
UNCLOS	loop265	loopsieved	0	30	80.518	-147.488	beaufort	71.25277824
UNCLOS	loop28	loopsieved	0	30	79.999	5.872666667	fram	166.2045602
UNCLOS	loop307	loopsieved	0	30	78.988	-167.8765	beaufort	49.6687631
UNCLOS	loop307blank	loopsievedblank	0	30	78.988	-167.8765	beaufort	46.85055936
UNCLOS	loop315	loopsieved	0	30	78.317	-164.2223333	beaufort	78.82599581
UNCLOS	loop325	loopsieved	0	30	79.255	-162.7133333	beaufort	22.90955641
UNCLOS	loop335	loopsieved	0	40	80.553	-159.8403333	beaufort	7.226415094
UNCLOS	loop353	loopsieved	0	30	81.853	-150.7106667	beaufort	6.07966457
UNCLOS	loop360	loopsieved	0	30	81.725	-146.9641667	beaufort	28.98922098
UNCLOS	loop370	loopsieved	0	30	77.872	-146.2525	beaufort	16.74633124
UNCLOS	loop370blank	loopsievedblank	0	30	77.872	-146.2525	beaufort	49.29110777
UNCLOS	loop41	loopsieved	0	30	82.23708	10.96276	fram	65.17311367
UNCLOS	loop5	loopsieved	0	30	72.522	13.6505	fram	23.85744235
UNCLOS	loop54	loopsieved	0	30	84.89	13.57166667	fram	93.21533923
UNCLOS	loop67	loopsieved	0	30	87.611	2.9775	pole	51.98233799
UNCLOS	loop77	loopsieved	0	30	89.044	-19.53016667	pole	59.1770097
UNCLOS	loop90	loopsieved	0	30	88.939	-54.0515	pole	54.90955641
UNCLOS	loop90blank	loopsievedblank	0	30	88.939	-54.0515	pole	78.36388935

2b) lengths

longitude	Fiber length (microns)
-53.79	1488
-139.69424	783
-124.98682	825
-60.79	405
-162.276	300
-152.0653	1743
-97.031	1760
-163.856	235
-94.216	933
13.6505	435
-144.7043	1148
-78.197	683
-81	1133
-139.9428	1113
-89.0363	640
-91.971	810
-150.023	780
-149.9902	730
-153.6673	860
8.8015	1555
8.793333333	970
-149.8928	1270
-164.2223333	930
-130.9431	595
5.872666667	1852
-140.5691667	577
13.57166667	783
-147.8465	905
-134.7785	3630
-134.745	220
-53.99066667	717
-54.0515	1410
48.47566667	2040

2c) Peak Ratio Index

longitude	PRI_index	station_code
-124.98682	-1.963635071	loop01
13.6505	-1.696928565	loop05
-153.6673	-1.49871682	loop107
48.47566667	-0.565665443	loop145
-134.7785	-0.916141186	loop165
8.8015	-0.458336132	loop17b
-69.99372022	-0.853011912	loop18
-147.8465	-0.656705026	loop203
-139.69424	-1.979322337	loop206
-140.5691667	-0.859131771	loop237
-164.2223333	-2.795641819	loop315
-140.275155	-2.24696604	loop40
13.57166667	0.919412987	loop54
-93.38445522	-2.205731089	loop90

-53.79	-0.499191472	oe01
-91.971	-0.131377466	oe10
-94.216	-0.672305127	oe14
-97.031	0.345501514	oe17

Supplementary Table 3 | Blanks

station	particle number	photo	fibre	fibre length	fibre width	colour	transparent
pblank1	1	1	N	NA	NA	blue	Y
pblank1	2	2	Y	1410	7	blue	N
pblank1	3	3	Y	170	6	purple	N
pblank1	4	4	Y	1750	22	brown	N
pblank1	5	5	Y	2785	12	clear	Y
pblank1	6	6	Y	140	10	purple	N
pblank1	7	7	Y	145	4	purple	N
pblank1	8	8	Y	550	10	blue	N
pblank2	1	1	Y	350	17	orange	Y
pblank2	2	2	Y	1220	17	purple	N
pblank2	3	3	Y	745	5	blue	N
pblank2	4	4	Y	600	18	orange	N
pblank2	5	5	Y	970	17	orange	N
pblank2	6	6	Y	135	18	orange	N
pblank2	7	6	Y	115	14	blue	N
pblank2	8	7	Y	6320	18	yellow	Y
pblank2	9	8	Y	5000	10	red	N
pblank2	10	9	Y	2180	16	dark	N
pblank2	11	10	Y	1245	6	orange	N
pblank2	12	11	Y	405	20	blue	N
pblank2	13	12	Y	305	10	orange	N
pblank2	14	12	Y	300	10	dark	N
pblank3	1	2	Y	1990	10	dark	N
pblank3	2	3	Y	130	7	dark	N
pblank3	3	4	Y	180	30	brown	N
pblank3	4	5	Y	715	30	clear	Y
pblank4	1	2	Y	145	16	orange	Y
pblank4	2	3	Y	990	20	blue	N
pblank4	3	4	Y	9600	8	blue	N
pblank4	4	5	Y	545	42	orange	N
pblank4	5	6	Y	790	12	blue	Y
pblank4	6	7	Y	1955	25	orange	N
pblank5	1	2	Y	560	10	clear	Y
pblank5	2	3	Y	625	33	orange	N
pblank5	3	4	Y	670	19	purple	Y
pblank5	4	5	Y	765	12	clear	Y
pblank5	5	6	Y	1035	14	clear	y
pblank5	6	7	Y	200	21	orange	N
pblank5	7	8	Y	300	25	brown	N
pblank5	8	9	Y	270	32	brown	N

pblank5	9	10	Y	300	26	yellow	Y
pblank5	10	11	Y	570	40	yellow	N
pblank5	11	12	Y	735	18	orange	N
pblank5	12	13	Y	1295	20	orange	N
pblank5	13	14	Y	940	10	red	N
pblank5	14	15	Y	410	8	clear	Y
pblank5	15	16	Y	375	9	clear	Y
pblank5	16	17	Y	1270	16	brown	Y
pblank5	17	18	Y	4220	36	yellow	Y
pblank5	18	19	Y	1065	13	clear	N
pblank5	19	20	Y	390	18	clear	N
pblank5	20	20	Y	925	18	clear	N
pblank6	1	2	Y	630	16	yellow	N
pblank6	2	3	Y	255	40	yellow	N
pblank6	3	4	Y	865	19	yellow	N
pblank6	4	5	Y	305	18	orange	N
pblank7	1	2	Y	170	10	orange	N
pblank7	2	3	Y	410	7	orange	N
pblank7	3	4	Y	900	12	orange	N
pblank7	4	5	Y	575	14	orange	N
pblank7	5	6	Y	950	15	orange	N
pblank8	1	3	Y	1290	22	clear	Y
pblank8	2	4	Y	90	6	orange	N
pblank8	3	5	Y	435	25	orange	N
pblank8	4	6	Y	175	20	orange	N
pblank8	5	7	Y	145	8	yellow	N
pblank8	6	8	Y	85	16	orange	N
